# Psychosocial and cognitive function in children with nephrotic syndrome: association with disease and treatment variables

**DOI:** 10.1186/1751-0759-7-10

**Published:** 2013-04-23

**Authors:** Panagiota Manti, George Giannakopoulos, Elena Giouroukou, Helen Georgaki-Angelaki, Constantinos J Stefanidis, Andromahi Mitsioni, Nikolaos Stergiou, Constantinos Mihas, George P Chrousos, Maria Alexandra Magiakou, Gerasimos Kolaitis

**Affiliations:** 1 Faculty of Health and Caring Professions, 2nd Department of Nursing, Technological Educational Institution of Athens, Athens, Greece; 2 Department of Child Psychiatry, “Aghia Sophia” Children’s Hospital, University of Athens Medical School, Athens 11527, Greece; 3 Department of Pediatric Nephrology, “Aghia Sophia” Children’s Hospital, Athens, Greece; 4 Department of Pediatric Nephrology, “P. & A. Kyriakou” Children’s Hospital, Athens, Greece; 5 Division of Endocrinology, Metabolism and Diabetes, 1st Department of Pediatrics, “Aghia Sophia” Children’s Hospital, University of Athens Medical School, Athens, Greece

**Keywords:** Children, Cognitive, Nephrotic syndrome, Psychosocial, Steroids

## Abstract

**Background:**

To investigate possible differences in emotional/behavioral problems and cognitive function in children with nephrotic syndrome compared to healthy controls and to examine the effect of disease-specific and steroid treatment-specific characteristics on the abovementioned variables.

**Methods:**

Forty-one patients with nephrotic syndrome (23 boys, age range: 4.4-15.2 years) and 42 sex- and age-matched healthy control subjects (20 boys, age range: 4.1-13.4 years) were enrolled in the study. Disease (severity, age of diagnosis, duration) and steroid treatment (total duration, present methylprednisolone dose and duration of present dose) data were collected. In order to assess children’s emotional/behavioral problems, the Child Behavior Checklist was administered. The Wechsler Intelligence Scale for Children – Third Edition was administered to assess Full-Scale, Verbal, and Performance intelligence quotient (IQ) scores.

**Results:**

The patients presented with more internalizing problems (*P* = 0.015), including withdrawal (*P* = 0.012) and somatic complaints (*P* = 0 .011), but not more anxiety/depression or externalizing problems. A significant association was found between severity of disease and somatic complaints (*P* = 0.017) as well as externalizing problems (*P* = 0.030). Years of illness were significantly more in those presenting with abnormal anxiety/depression (*P* = 0.011). Duration of steroid medication was significantly higher among those presenting with abnormal anxiety/depression (*P* = 0.011) and externalizing problems (*P* = 0.039). IQ was not associated significantly with disease or steroid treatment variables.

**Conclusions:**

Psychosocial factors and outcomes may be important correlates of children’s nephrotic syndrome and potential targets of thorough assessment and treatment.

## Background

It has been recognized that children with nephrotic syndrome often experience significant emotional and behavioral problems, especially those with frequent relapses, steroid dependence, steroid resistance, or maternal distress [1]. However, the evidence is rather limited. Higher levels of anxiety, depression, aggression, and inattention in children with nephrotic syndrome compared to healthy controls have been reported in previous studies [2-7]. A prospective study [8] found negative behavioral changes in children with nephrotic syndrome during periods of relapse. Nevertheless, it is very difficult to differentiate a disease-specific effect as opposed to a treatment effect on emotional and behavioral health, since all children with nephrotic syndrome are treated with steroids at diagnosis. Studies in pediatric patients treated with oral, inhaled, and intravenous steroids indicate that adverse psychological side effects can occur at any point during treatment [8-13]. It has been suggested that children with nephrotic syndrome may be particularly susceptible to the side effects of steroids because of the increased free serum prednisolone levels measured during periods of hypoalbuminemia [8]. One study [2] showed that children receiving high-dose oral steroid treatment for nephrotic syndrome experienced worsening behavioral changes. However, another larger study [4] did not detect any association between steroid treatment and behavior abnormality. In the present study, we tried to investigate possible differences in emotional/behavioral problems and cognitive function in children with nephrotic syndrome compared to healthy controls and to examine the effect of disease-specific and treatment-specific characteristics.

## Methods

### Participants and procedures

Forty-one patients with nephrotic syndrome (23 boys, age range: 4.4-15.2 years) and 42 sex- and age-matched healthy control subjects (20 boys, age range: 4.1-13.4 years) were enrolled in the study. All patients were diagnosed at the Pediatric Departments of “Aghia Sophia” and “P. & A. Kyriakou” Children’s Hospitals, Athens, Greece, and followed-up as outpatients at regular intervals since diagnosis in the Pediatric Nephrology Departments of either hospital. All were on steroid treatment for at least 4 consecutive months before entry. The 4-month period was chosen arbitrarily as a determinant of chronic treatment. The control subjects were healthy children evaluated for growth and puberty in the Division of Endocrinology, Metabolism, and Diabetes of the First Department of Pediatrics of “Aghia Sophia” Children’s Hospital. The study was approved by the Scientific and Ethics Committees of “Aghia Sophia” and “P. & A. Kyriakou” Children’s Hospitals, and all parents provided written informed consent before entry of their children in the study.

### Measures

Nephrotic syndrome was defined according to the *International Study of Kidney Diseases in Children* (ISKDC) [14]. Relapse was defined as proteinuria (Albustix > 2+) for more than 3 consecutive days. Frequent relapsers were defined according to the ISKDC criteria (more than 2 relapses in the initial 6 months after presentation or > 4 per year during follow-up) [15]. Steroid dependency was defined according to *Arbeitsgemeinschaft fur PaediatrischeNephrologie* (APN) as at least 2 relapses during alternate-treatment with prednisone or within 2 weeks of cessation [16]. The disease was characterized as mild (< 2 relapses per year without alternative treatment other than steroids), moderate (2–4 relapses per year with alternative treatment) or severe (> 4 relapses per year ± alternative treatment).

All patients and healthy control subjects were informed about the study and an appointment was arranged for the day of evaluation. The nephrologists provided the patients’ medical records with the detailed history. On the day of evaluation, a detailed history was obtained and a clinical examination was performed. The patients and their parents were asked in detail about the disease characteristics, the medications and doses given, the remission periods and number of relapses, and the present steroid regimen and dose used. The control subjects were also asked about their previous history, and serious diseases or previous chronic treatment with steroids represented exclusion criteria. On clinical examination, systolic and diastolic blood pressure, height, weight, and pubertal stage were precisely determined in all children. The body mass index (BMI) was calculated using the formula: weight (kilograms)/height (meters) [2]. Pubertal staging was based on Tanner’s criteria. Waist and hip circumferences were also recorded and blood pressure was measured. Waist to hip ratio (WHR) Standard Deviation score (SDS) was calculated using Dutch age references [17]. Greek longitudinal normative data were used as standards for the calculation of height, weight and BMI SDS [18,19]. Parental educational level was collected through parents’ reports using the International Standard Classification of Education (ISCED) [20]. The original 7 educational levels were codified in the analysis into 3 categories: primary school (categories 0, 1 and 2), secondary school (categories 3 and 4) and university degree (categories 5 and 6).

In order to assess children’s emotional/behavioral problems, the Child Behavior Checklist (CBCL) [21,22], a 113-item parent-report checklist, was administered. Parents rate the frequency of specific behaviors in their children during the previous 6 months. The CBCL yields the following factors: anxious/depressed, withdrawn, somatic complaints, social problems, thought problems, attention problems, delinquent behavior, aggressive behavior, internalizing problems, externalizing problems, and total problems. Higher T-scores reflect poorer functioning, with T-scores < 65 indicating normal problems, 65–69 borderline problems and > 69 abnormal problems.

The Wechsler Intelligence Scale for Children – Third Edition (WISC-III) was administered to assess Full-Scale, Verbal and Performance intelligence quotient (IQ) scores [23]. IQ scores for children younger than 6 years old were measured by the Wechsler Preschool and Primary Scale of Intelligence – Third Edition (WPPSI-III) [24].

### Data analysis

All continuous variables are presented as median ± interquartile range (75th -25th percentile, IQR) since the majority of them deviated from normal distribution, according to the results of Shapiro-Wilk test for normality. All categorical variables are presented as absolute and relative (percentage) frequencies. The Mann–Whitney *U* test was used in order to evaluate the differences in continuous variables between different groups. Pearson’s chi-square or Fisher’s exact statistics were used in order to investigate potential associations between categorical variables. Spearman’s correlation coefficient was used in order to assess the associations between continuous variables. All tests were two-sided at a significance level of *P* < .05. Data were analyzed using STATA™ statistical software (Version 9.0, Stata Corporation, College Station, TX 77845, USA).

## Results

The disease and steroid treatment characteristics of the patients are depicted in Table 1. Thirty-eight of the 41 patients (92.7%) were on alternate-day steroid treatment, 24/41 (58.5%) had steroid-dependent NS, and 22/41 (53.7%) were also on other immunosuppressive drugs (five patients were on cyclosporine, four on cyclophosphamide, two on MMF, one on ergamisole, and 10 patients were on combined immunosuppressive treatment with the previously determined regimens). Fourteen, 16, and 11 patients had mild, moderate and severe disease, respectively.

**Table 1:** Disease and steroid treatment characteristics of the patient group

		**Patients**	
		**n (%)**	
Severity of disease	Mild	14 (34.1)	
	Moderate	16 (39.0)	
	Severe	11 (26.8)	
		**Median**	**IQR**
Age at diagnosis (ys)		3.50	1.90
Duration of illness (ys)		3.00	4.60
Total duration of steroid medication (months)		31.00	44.00
Present methylprednisolone dose (mg/kg/day)^*^		0.16	0.19
Duration of present dose (days)**		38.00	99.00

*IQR*, Interquartile range (75th-25th percentile).

^*^18/41 were on methylprednisolone and 23/41 on prednisolone treatment. The dose was homogenized using the appropriate equivalence.

** The steroid dose was often changed for better outcome.

The distribution of clinical and demographic characteristics by group is shown in Table 2. Almost 1 in 5 parents (19.5%) of children with nephrotic syndrome had a low educational level while all parents of the children in the control group had at least a medium educational level (*P* < 0.001). Children with nephrotic syndrome had significantly higher BMI (*P* < 0.001), WHR (*P* = 0.002) and systolic pressure SDS (*P* < 0.001) as well as significantly lower body height SDS (*P* < 0.001) than controls, presumably due to the steroid treatment.

**Table 2:** Clinical and demographic characteristics of the patient and control groups

		**Group**				
		**Patients**		**Controls**		
		**n (%)**		**n (%)**		***P***
Sex	Boys	23 (56.1)		20 (47.6)		0.440
	Girls	18 (43.9)		22 (52.4)		
Nationality	Greek	35 (85.4)		39 (92.9)		0.313
	Other	6 (14.6)		3 (7.1)		
Parental education	Low	8 (19.5)		0 (0.0)		<0 .001
	Medium	28 (68.3)		21 (52.5)		
	High	5 (12.2)		19 (47.5)		
Pubertal stage	Preadolescence	35 (85.4)		31 (73.8)		0.192
	Adolescence	6 (14.6)		11 (26.2)		
		**Median**	**IQR**	**Median**	**IQR**	***P***
Age (ys)		7.08	4.75	9.04	4.00	0.146
WHR SDS		0.76	0.92	0.04	0.83	0.002
BMI SDS		0.55	1.29	−0.32	1.30	<0.001
Body weight SDS		−0.05	1.27	−0.26	1.58	0.439
Body height SDS		−1.18	1.31	0.17	1.71	<0.001
Systolic blood pressure SDS		1.15	1.36	−0.31	1.60	<0.001
Diastolic blood pressure SDS		0.64	0.90	0.47	1.40	0.436

*IQR*, Interquartile range (75th-25th percentile), WHR = Waist to hip ratio, SDS = Standard deviation score, *BMI*, Body mass index.

A positive trend between CBCL factors such as withdrawn, somatic complaints, thought, internalizing, and total problems and the presence of disease was noticed (Table 3), since a significantly higher frequency of abnormality (T-score > 69) was noticed in the patient rather than in the control group in the aforementioned variables (9.8% vs. 0%, 12.8% vs. 0%, 23.1% vs. 2.4%, 25.0% vs. 2.5%, 32.1% vs. 7.7%, respectively). Verbal, performance and full-scale IQ were significantly lower in the children with nephrotic syndrome compared to the controls (114.0 ± 26.0 vs. 117.0 ± 20.0, 110.0 ± 13.5 vs. 114.0 ± 23.0, 110.5 ± 19.0 vs. 120.0 ± 26.0, respectively).

**Table 3:** Emotional/behavioral problems and intelligence quotient (IQ) scores of the patient and control groups

		**Group**				
		**Patients**		**Controls**		
	**CBCL T-scores**	**n (%)**		**n (%)**		***P***
Anxious/depressed	<65	29 (78.4)		37 (90.2)		0.330
	65-69	5 (13.5)		2 (4.9)		
	>69	3 (8.1)		2 (4.9)		
Withdrawn	<65	31 (75.6)		40 (97.6)		0.012
	65-69	6 (14.6)		1 (2.4)		
	>69	4 (9.8)		0 (.0)		
Somatic complaints	<65	31 (79.5)		41 (97.6)		0.011
	65-69	3 (7.7)		1 (2.4)		
	>69	5 (12.8)		0 (.0)		
Social problems	<65	30 (81.1)		40 (95.2)		0.094
	65-69	6 (16.2)		2 (4.8)		
	>69	1 (2.7)		0 (.0)		
Thought problems	<65	29 (74.4)		38 (92.7)		0.008
	65-69	1 (2.6)		2 (4.9)		
	>69	9 (23.1)		1 (2.4)		
Attention problems	<65	36 (97.3)		41 (97.6)		0.999
	65-69	1 (2.7)		1 (2.4)		
	>69	0 (.0)		0 (.0)		
Delinquent behavior	<65	28 (84.8)		32 (76.2)		0.289
	65-69	3 (9.1)		9 (21.4)		
	>69	2 (6.1)		1 (2.4)		
Aggressive behavior	<65	30 (78.9)		38 (92.7)		0.243
	65-69	3 (7.9)		1 (2.4)		
	>69	5 (13.2)		2 (4.9)		
Internalizing problems	<65	22 (61.1)		32 (80.0)		0.015
	65-69	5 (13.9)		7 (17.5)		
	>69	9 (25.0)		1 (2.5)		
Externalizing problems	<65	20 (62.5)		30 (73.2)		0.513
	65-69	5 (15.6)		6 (14.6)		
	>69	7 (21.9)		5 (12.2)		
Total problems	<65	18 (64.3)		31 (79.5)		0.027
	65-69	1 (3.6)		5 (12.8)		
	>69	9 (32.1)		3 (7.7)		
		**Median**	**IQR**	**Median**	**IQR**	***P***
Verbal IQ		114.00	26.00	117.00	20.00	0.025
Performance IQ		110.00	13.50	114.00	23.00	0.015
Full-scale IQ		110.50	19.00	120.00	26.00	0.005

*IQR*, Interquartile range (75th-25th percentile), *IQ*, Intelligence quotient.

In an attempt to adjust for the significantly different distribution of educational level between patients and controls, all of the variables that were found to differ significantly (withdrawn, somatic complaints, thought problems, internalizing problems, total problems, verbal, performance and full-scale IQ) were re-evaluated separately for each educational level (low level was not considered since none of the controls reported such). In those children whose parents had a medium educational level, no significant difference was found in any variable except full-scale IQ (patients: 110.5 ± 20.5, controls: 118.0 ± 20.0, *P* = 0.039). When the educational level of the parents was high, borderline/abnormal emotional/behavioral problems that were significantly more frequent among patients compared to controls were withdrawn (patients: 40%/0%, controls: 0%/0%, *P* = 0.036), somatic complaints (patients: 20%/20%, controls: 0%/0%, *P* = 0.036), and internalizing problems (patients: 0%/60%, controls: 11.1%/5.6%, *P* = 0.040).

Regarding severity of disease and child emotional/behavioral problems, a significant association was found between somatic complaints and severity of disease. Forty percent of those having a severe disease reported CBCL somatic complaints T-scores > 69, while the corresponding relative frequency was 7.7% in those having a mild disease (*P* = 0.017). Those reporting CBCL externalizing problems T-scores > 69 were 33.3% of those having a severe disease, 23.1% of those having a moderate disease, and 15.4% of those having a mild disease, underlining the significant association between the two aforementioned variables (*P* = 0.030). None of the other CBCL factors (anxious/depressed, withdrawn, social problems, thought problems, attention problems, delinquent behavior, aggressive behavior, internalizing problems, and total problems) were significantly correlated with severity of disease.

From all the factors of the CBCL, years of illness differed significantly only in those reporting anxiety/depression T-scores > 69 (7.8 ± 3.3) compared to those reporting a T-score < 65 (3.0 ± 3.9, *P* = 0.011). None of the child emotional/behavioral problems as reported in CBCL T-scores associated significantly with steroid dose/kg/day. Duration of steroid medication differed among the T-score categories of anxiety/depression (< 65: 31.0 ± 51.0, 65–69: 12.0 ± 2.0, > 69: 94.0 ± 51.0, *P* = 0.011) and among those of externalizing problems (< 65: 31.0 ± 51.0, 65–69: 12.0 ± 2.0, > 69: 94.0 ± 51.0, *P* = 0.039).

Verbal, performance and full-scale IQ did not differ significantly among the categories of severity of disease (mild, moderate, and severe) and were not significantly correlated with years of illness, steroid dose/kg/day, or duration of steroid medication.

## Discussion

Our findings suggest that children with nephrotic syndrome may experience higher levels of emotional/behavioral problems related to disease- and treatment-specific variables. Based on the responses of the CBCL, the patients presented with more internalizing problems, such as withdrawal and somatic complaints than healthy controls. However, unlike previous reports [2-7], no marked differences were found between the two groups regarding anxiety/depression or externalizing problems.

It is quite difficult to differentiate the effects of treatment vs. the disease itself on behavior. When in remission, the child with nephrotic syndrome may appear physically well, but medical insistence on regular monitoring for proteinuria is an ever-present reminder that there is an underlying chronic medical condition, which may potentially relapse. The negative impact of chronic illness on emotional/behavioral development is well documented and epidemiologic studies estimated that children with chronic illnesses are 2 to 2.4 times more likely to develop diagnosable psychiatric and behavioral disorders [7]. Children in the present sample with severe disease seemed to present more somatic complaints and externalizing problems than did those with mild or moderate disease. Similar results have been previously reported, with frequent relapses being associated with somatic complaints and externalizing/aggressive behavior [1,5,8]. Moreover, the duration of the illness was associated with more anxiety/depression symptoms. This finding is in line with previous observations identifying duration of the disease as a strong predictor of emotional problems [5]. The prolonged need to take medications, frequent contacts with medical professionals, interruptions in schooling and everyday activities, and parents’ anxious concerns about the course of the illness may be mechanisms through which the illness duration increases children’s anxiety and depression symptoms.

Among the treatment-specific variables examined in the present analysis, duration of steroid treatment was found to be significantly related to children’s anxiety/depression and externalizing problems. This finding may reflect the effect of the extended exposure to steroids on the children’s physical appearance and fitness, as it was reflected in the significant differences in the BMI, height, and WHR between patients and controls in the present sample. Changes in these crucial dimensions of children’s self-image can affect their self-esteem and their peer relationships (e.g. facing social exclusion or bullying) leading to anxiety/depression and behavior problems. Moreover, it could be hypothesized that anxiety/depression problems may be partly induced by steroid effects on brain regions (i.e. hippocampus and amygdala) involved in mood regulation and in which corticosteroid receptors are densely located. Also, externalizing problems, which were here significantly associated both with duration of steroid medication and severity of disease, may be attributable to a trend of increased emotional liability and aggressive behavior in nephrotic children under steroid medication [8]. The abovementioned findings are in line with previous literature indicating that insomnia, tearfulness, irritability, argumentative behavior, and tiredness are more common in children on steroids than controls [8-13]. On the other hand, the present study did not manage to find any associations between steroid dose and emotional/behavioral abnormalities, which have been reported elsewhere [2,5]. Moreover, previous reports have yielded conflicting findings with some studies showing no associations [25,26] and other studies indicating diminished verbal memory in high steroid dose (61.5 mg prednisolone/day) groups compared to low dose (3–7 mg prednisolone/day) groups [11,13]. In the present samples no associations were found between steroid treatment characteristics and IQ scores, with the exception of patients with medium parental education level that showed lower full-scale IQ scores compared to controls. However, it is difficult to judge these differences, since low and high educational levels are under-represented in the present sample, compared to Greek community samples [27]. Furthermore, it should be mentioned that the steroid doses administered at the time of evaluation in the present sample were mostly low, since most patients were in remission. Overall, the present study did not manage to draw a definite conclusion regarding the effect of steroids on the regulation of behavior, mood, and cognitive functions among children with nephrotic syndrome. The fact that, in the present sample, no differences were observed between patients and healthy controls regarding anxiety and externalizing problems, in particular, although these problems were associated with medication and disease characteristics in the patient group, needs to be interpreted with caution. This discrepancy may suggest that the absence of any differences concerning anxiety and externalizing problems may be due to the fact that the sample size was rather small and/or the administered steroid doses were mostly low, as previously mentioned. Further research with a larger sample receiving higher steroid doses (e.g. with patients being in relapse) is needed in order to enhance the validity of this examination.

The present study contributes to previous research mainly in two ways. First, it examined the associations of several psychosocial factors and outcomes with pediatric nephrotic syndrome providing a comprehensive picture of this complex interplay. Second, it shed some light on the relationship of the disease and treatment characteristics with children’s cognitive functions as measured through verbal and performance IQ scales, an issue that was missing in pediatric nephrotic syndrome literature. The findings reported here can offer some useful implications. Addressing the psychosocial needs of children in the course of nephrotic syndrome seems to be of importance in any effective preventive and therapeutic intervention. Moreover, the role of prolonged exposure to the disease itself and its treatment seem to be a possible target for future research and psychosocial interventions.

The present findings should be interpreted in the context of some limitations. First, the study was based only on parent-reported children’s emotional/behavioral problems that limit the interpretation of the results. Second, the study did not examine the effect of socioeconomic status on the reported differences, threatening generalizability of results. Moreover, parents with low educational level were under-represented in the study. Similarly, no definite conclusions can be drawn regarding the effect of parental education on the observed differences, since parents with low educational level were under-represented in the present study. The control group, in particular, did not include parents with low education level, while the latter represents more than one third of cases in nation-wide community samples. This possible selection bias may be due to the fact that the control group was recruited from a health service for growth and development evaluation, where families with low educated parents more rarely seek help [27]. In addition, the sample size was rather small, leading to a low statistical power. Adjustment for multiple comparisons was not performed due to the fact that our sample was relatively small and many variables had to be tested. Last, the cross-sectional design of the study did not allow us to examine variations over time or make causal inferences. Regardless of these limitations, the present study clearly suggests that psychosocial factors and outcomes may be important correlates of children’s nephrotic syndrome and potential targets of thorough assessment and treatment.

## Conclusions

Children with nephrotic syndrome presented with more withdrawal problems and somatic complaints, but not more anxiety/depression or externalizing problems compared to their healthy counterparts. The severity of disease was positively associated with more somatic complaints as well as externalizing problems. Years of illness were significantly more in those presenting with abnormal anxiety/depression Duration of steroid medication was significantly higher among those presenting with abnormal anxiety/depression and externalizing problems. Addressing the psychosocial needs of children in the course of nephrotic syndrome seems to be of importance in any effective preventive and therapeutic intervention. The role of prolonged exposure to the disease itself and its treatment seem to be a possible target for future research and psychosocial interventions.

## Competing interests

The authors declare that they have no competing interests.

## Authors’ contributions

PM conceptualized and carried out the study, and drafted the manuscript. GG assisted with the study conceptualization and drafted the manuscript. EG, HG-A, CJS, AM, NS assisted with the study. CM performed the statistical analysis. GPC conceptualized and coordinated the study. MAM and GK conceptualized and coordinated the study, and drafted the manuscript. All authors read and approved the final manuscript.
